# Metformin’s Modulatory Effects on miRNAs Function in Cancer Stem Cells—A Systematic Review

**DOI:** 10.3390/cells9061401

**Published:** 2020-06-04

**Authors:** Bartosz Malinowski, Nikola Musiała, Michał Wiciński

**Affiliations:** Department of Pharmacology and Therapeutics, Faculty of Medicine, Collegium Medicum in Bydgoszcz, Nicolaus Copernicus University, M. Curie 9, 85-090 Bydgoszcz, Poland; nicole.musiala@gmail.com (N.M.); wicinski4@wp.pl (M.W.)

**Keywords:** cancer stem cells, metformin, miRNA

## Abstract

Cancer stem cells (CSCs) have been reported in various hematopoietic and solid tumors, therefore, are considered to promote cancer progression, metastasis, recurrence and drug resistance. However, regulation of CSCs at the molecular level is not fully understood. microRNAs (miRNAs) have been identified as key regulators of CSCs by modulating their major functions: self-renewal capacity, invasion, migration and proliferation. Various studies suggest that metformin, an anti-diabetic drug, has an anti-tumor activity but its precise mechanism of action has not been understood. The present article was written in accordance to the PRISMA (Preferred Reporting Items for Systematic Reviews and Meta-Analyses) guidelines. We systematically reviewed evidence for metformin’s ability to eradicate CSCs through modulating the expression of miRNAs in various solid tumors. PubMed and MEDLINE were searched from January 1990 to January 2020 for in vitro studies. Two authors independently selected and reviewed articles according to predefined eligibility criteria and assessed risk of bias of included studies. Four papers met the inclusion criteria and presented low risk bias. All of the included studies reported a suppression of CSCs’ major function after metformin dosage. Moreover, it was showed that metformin anti-tumor mechanism of action is based on regulation of miRNAs expression. Metformin inhibited cell survival, clonogenicity, wound-healing capacity, sphere formation and promotes chemosensitivity of tumor cells. Due to the small number of publications, aforementioned evidences are limited but may be consider as background for clinical studies.

## 1. Introduction

Cancer stem cells (CSCs) are a subpopulation of cancer cells that have the ability to self-renew, differentiate into different cell types and to arrest in the G0 phase. Therefore, CSCs may be the main reason for the failure of cancer treatment, by causing metastasis, recurrence and resistance to therapy [1,2]. In the 1990s, CSCs were identified in acute myeloid leukemia (AML) [3,4]. Bonnet and Dick [4] described CD34^+^CD38^−^ leukemic cells that could initiate AML in NOD/SCID (non-obese diabetic/severe combined immunodeficiency) mice. Further research has provided evidence of the presence of CSCs in many solid tumors, for example, breast [5], ovarian [6,7] and pancreatic [8,9].

Since the first studies on CSCs’ existence, the expression of cell surface markers has been used to isolate and identify CSCs, differentiating them from many types of cancers [4,5,6]. There are plenty of common or unique surface markers that have been associated with solid or hematopoietic tumors, for example, CD34^+^CD38^−^ for AML [4]; CD44^+^CD24^−^/lowLin- [5] and ALDH+ [10] for breast cancer; CD44^+^ [11], CD44^+^α2β1^+^ [12] and ALDH+ [13] for prostate cancer; CD44^+^CD117^+^ [7], CD24^+^ [14], ALDH+ [15] and CD133^+^ [16] for ovarian cancer. Heterogeneity of CSCs are complex and it is still unclear if phenotypically heterogeneous CSCs populations are also functionally different [17].

It is of great importance to understand the characteristics of CSCs. Like normal stem cells (NSCs), a major property of CSCs is their ability to self-renew [18]. In NSCs there are many signaling pathways that are strictly controlled, for example, Wnt/β-catenin, Notch, Hedgehog (Hh) and B-cell-specific Moloney murine leukemia virus integration site 1 (BMI1). However, due to epigenesis those self-renewal pathways (SRPs) are deregulated in CSCs [19]. It is still poorly understood how CSCs are regulated at the molecular level [18,19].

Recent studies of microRNAs (miRNAs) have introduced their new major role in regulatory mechanisms in CSCs [20]. miRNAs are small molecules (21–25 nucleotides long), that belong to a class of non-coding RNAs. Through binding to the 3′-untranslated regions (3′-UTR) of target mRNAs, miRNAs regulate gene expression [21]. Various studies indicate that miRNAs are involved in a wide range of cell functions, such as development, proliferation, differentiation, apoptosis and self-renewal [22,23]. Those evidences link miRNAs to the regulatory mechanisms at the molecular level of NSCs and CSCs. Moreover, through the regulation of the key biological properties of CSCs, it has become evident that miRNAs are involved in tumorigenesis [23].

Traditional cancer treatment may not affect CSCs due to their mechanism of drug resistance. CSCs are mostly arrested in the G0 phase; they express ATP-binding cassette (ABC) transporters (ABCB1, ABCC1, ABCG2) and prevent cancer cells from apoptosis. The ABC transporters use energy from ATP hydrolysis to translocate various substances across the cell membrane. Overexpression of ABC proteins is the main protective mechanism for CSCs from various agents. Additionally, aldehyde dehydrogenase (ALDH), a cytosolic enzyme that oxidizes aldehydes, enhances resistance to chemotherapy and radiotherapy through protecting CSCs from oxidative stress. The drug-resistance characteristics of CSCs play an essential role in cancer progression and relapse [24,25]. It is crucial to develop new therapeutic strategies that target CSCs. Recent studies have been focused on a well-known drug—metformin—that may play a major role in regulation of miRNAs functions [26]. Metformin is an anti-hyperglycemic agent that is widely used for treating patients with type-II DM (diabetes mellitus) and also with polycystic ovarian syndrome (PCOS). Despite the widespread use of metformin, the molecular mechanisms of action of the drug are still largely debated [27,28]. Metformin acts through inhibition of the complex I of the mitochondrial respiratory chain which increases the cellular AMP:ATP ratio [29]. AMP-activated protein kinase (AMPK) is a key enzyme of energy homeostasis that is activated through change in the AMP:ATP ratio [30,31]. Metformin-mediated AMPK activation results in down-regulation of hepatic gluconeogenesis and up-regulation of glucose intake in peripheral tissue [31]. Interestingly, numerous studies bring a new possibility in metformin usage. It is considered, that metformin may function as an anti-tumor agent through regulation of miRNAs and CSCs [26,28].

Therefore, in this systematic review recent evidence of metformin influence on miRNAs and CSCs regulation in solid tumors will be discussed and summarized.

## 2. Methods

### 2.1. Data Sources and Searches

Two independent authors searched PubMed and MEDLINE for all published results between January 1990 and January 2020 on metformin influence on cancer stem cells through regulation of miRNAs. The following search terms were used: "neoplastic stem cells"(MeSH Terms) OR "neoplastic"(All Fields) AND "stem"(All Fields) AND "cells"(All Fields) OR "neoplastic stem cells"(All Fields) OR "cancer"(All Fields) AND "stem"(All Fields) AND "cells"(All Fields) OR "cancer stem cells"(All Fields)) AND "metformin"(MeSH Terms) OR "metformin"(All Fields) AND "micrornas"(MeSH Terms) OR "micrornas"(All Fields) OR "mirna"(All Fields) OR "miR"(All Fields). Investigators also searched the bibliographies of relevant articles.

### 2.2. Eligibility Criteria

Present systematic review was conducted in accordance with PRISMA (Preferred Reporting Items for Systematic Reviews and Meta-analyses) statement.

The PICO criteria:

Population: cells from human solid tumors that exhibit CSCs characteristics
Intervention: metformin
Comparison: cells without metformin treatment
Outcome: changes in miRNAs expression; inhibition of cell proliferation, migration, invasion and self-renewal capacity; inhibition of sphere formation

All published studies were only included if they were written in English and performed in in vitro experiments. However, included papers could additionally perform in vivo studies on mice. We also accepted articles that compared mechanism of action of metformin with other interventions that regulate miRNAs expression. Clinical trials were excluded from the present paper.

### 2.3. Study Selection

Two investigators (N.M., B.M.) independently reviewed each study’s title and abstract according to the prespecified eligibility criteria. Abstracts of interest were included for full-text analysis. Afterwards, two authors analyzed all full-text articles and rejected those that did not meet the aforementioned PICOs criteria. Any inconsistencies between the two reviewers were resolved by discussion with a supervisor (M.W.).

### 2.4. Data Collection Process and Data Items

All included articles were analyzed independently by the two authors (N.M., B.M.). The abstracted information included author names, year of publication, study design, cell line, animal model, intervention, dose of intervention, type of miRNA and main outcomes. The supervisor (M.W.) checked the abstracted information and resolved any disagreements.

### 2.5. Data Synthesis Analysis

Two investigators (N.M., B.M.) independently assessed the risk of bias in selected studies using predefined criteria. However, there is no standard risk-of-bias tool for in vitro studies; so methodological studies by criteria developed in the systematic reviews of in vitro studies (Table 1 and Table 2) were assessed [32]. Two authors (N.M., B.M.) independently categorized included studies as “low”, “moderate” or “high” quality. Any disagreements were resolved through discussion.

## 3. Results

### 3.1. Study Selection

**Figure 1:**
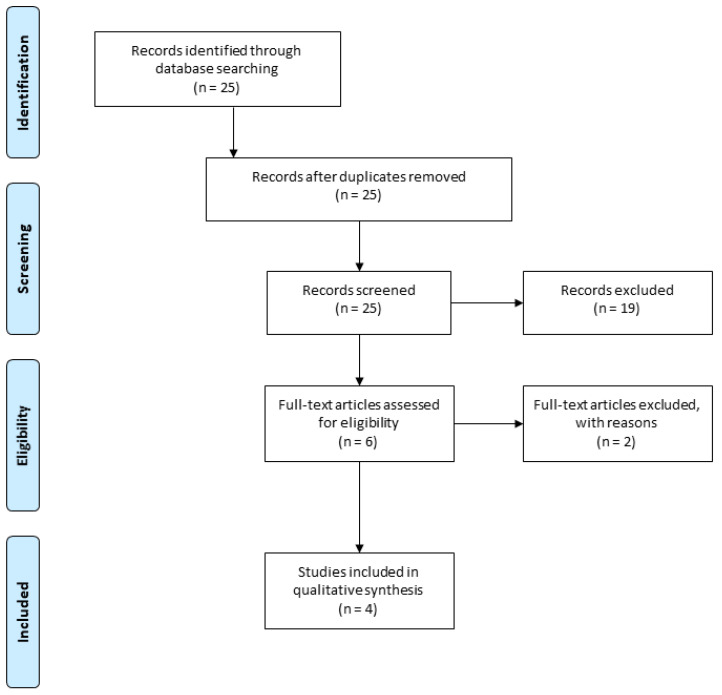
The Preferred Reporting Items for Systematic Reviews and Meta-Analyses (PRISMA) flow chart.

The electronic search from aforementioned databases revealed 25 articles in English. Of these, 19 were excluded after reading the title and abstract. The remaining 6 articles were included for full-text screening. Afterwards, 4 publications were included as they meet the inclusion criteria for this systematic review (Figure 1).

### 3.2. Study Characteristics

Characteristics of included studies are presented in Table 3. All articles were published between 2011 and 2019 and were written in English [33,34,35,36]. Studies have been carried out on two types of cancers: breast cancer [33,34,36] and pancreatic cancer [35]. The studies were conducted in the USA (*n* = 1) [35], Spain (*n* = 1) [36], Japan (*n* = 1) [34] and in China (*n* = 1) [33]. All four studies were performed in vitro [33,34,35,36], three of them also involved in vivo studies on animals (female BALB/c nude mice [33]; female NON/SCID mice [34]; female CB17/SCID mice [35]). Two studies analyzed cancer tissues from patients who underwent primary breast surgery for stage I-III [33] or II-III [34] invasive breast carcinoma. All studies used metformin as an intervention [33,34,35,36], one of them also used transforming growth factor β 1 (TGFβ1) with or without metformin [36]. The included papers examined the effects of metformin on expression of various miRNAs in cancer cells: microRNA-708 (miR-708) in breast cancer [33]; microRNA-27b (miR-27b) in breast cancer [34]; let-7a, microRNA-181a (miR-181a), and microRNA-96 (miR-96) in breast cancer [36]; let-7 family, microRNA-200 family (miR-200), microRNA-101 (miR-101) and microRNA-26a in pancreatic cancer [35]. Additionally, three articles showed the effect of metformin on the mRNA expression of CSCs marker genes [33,34,35]. Two papers examined the inhibition of spheres formation in cells treated with metformin [35,36].

### 3.3. Quality and Risk of Bias

All included papers were analyzed for risk of bias (Table 4 and Table 5). Three of them were considered “high” quality of evidence [33,34,35]. One study used only one cell line (MCF-7 cells) and was considered “moderate” quality [36]. Therefore, four included articles were considered as significant in reporting a potential effect of metformin on regulation of miRNAs expression and CSCs functions [33,34,35,36].

### 3.4. Results of Studies

#### 3.4.1. miRNAs Expression in Tumors

Tan et al. [33] analyzed miR-708 expression in the following cells derived from MDA-MB-231 and MCF-7 cells: spheres and adherent cells; non CD44^+^/CD24^−^ and CD44^+^/CD24^−^ population; cells treated with miR-708 knockdown or not; chemo resistant cell lines MCF-7ADR. miR-708 expression decreased significantly in mammospheres, CD44^+^/CD24^−^ population and in MCF-ADR cells. Moreover, cells treated with miR-708 knockdown showed enhancement of the mammospheres formation ability. Direct target of miR-708 has been identified as CD47. Additionally, overexpression of miR-708 or downregulation of CD47 induced sensitivity of MDA-MB-231 cells to docetaxel and increased the phagocytosis in all four cell lines [33]. Takahasi et al. [34] showed that downregulation of miR-27b induces drug resistance through formation of the SP fraction (side-population cells) of MCF-7 and ZR75-1 cells. Reduction of SP fraction occurs as a result of miR-27b suppression of the ectonucleotide pyrophosphatase/phosphodiesterase 1 (ENPP1) gene that leads to the inhibition of the expression and cell surface localization of ATP-binding cassette super-family G member 2 (ABCG2) transporter. Moreover, they confirmed that downregulation of miR-27b is associated with the generation of the high tumor seeding ability and chemoresistance population of luminal-type breast cancer cells, CD44^+^/CD24^−^ [34]. Bao et al. [35] examined the role of miR-26a, let-7b and miR-200b in pancreatic cancer cells. In MiaPaCa-2 cells, transfection of miR-26a precursor increased relative expression of miR-26a which caused a decrease in levels of enhancement of zeste homolog 2 (EZH2) and epithelial cell adhesion molecule (EpCAM) proteins and mRNA levels of EZH2, EpCAM, Oct4 and Notch-1. Additionally, investigators demonstrated that re-expression of let-7b and miR-26a decreased the formation of pancreatospheres in MiaPaCa-2 cells [35]. The results discussed above confirm that some miRNAs inhibit major properties of CSCs, such as drug resistance and self-renewal ability (Table 6) [33,34,35].

#### 3.4.2. Metformin Molecular Targets

All studies included for systematic review have analyzed the impact of metformin on selected miRNAs expression (Table 7). Tan et al. [33] demonstrated that in those cells treated with metformin, there was a significant increase of miR-708 expression and decrease of CD47 mRNA expression. Takahasi et al. [34] reported that metformin induced miR-27b-mediated suppression of ENPP1. Bao et al. [35] showed that metformin treatment increased the relative expressions of let-7a, let-7b, let-7c, miR-26a, miR-101, miR-200b and miR-200c in pancreatospheres. It was also found that metformin decreased the expressions of Oct4, Notch-1, EZH2 and Nanog mRNAs in pancreatospheres. Additionally, metformin inhibited the expression of CD44 and EpCAM in pancreatospheres [35]. Oliveras-Ferraros et al. [36] reported that metformin increased let-7A expression, downregulated TGFβ1-induced upregulation of miRNA-181a and suppressed TGFβ1-induced downregulation of miR-96.

#### 3.4.3. Impact of Metformin on Major CSCs Functions

Bao et al. [35] demonstrated that metformin decreased cell survival, clonogenicity, wound-healing capacity in all cell lines and invasion in parental MiaPaCa-2 and its tumor sphere cells. Moreover, it was also found that metformin either alone or in combination with difluorinated curcumin (CDF) inhibited the self-renewal ability of CSCs in primary and secondary pancreatospheres of all cell lines [35]. Authors showed that long-term metformin treatment decreased the formation of pancreatospheres induced by CSC-like cells [35]. Oliveras-Ferraros et al. [36] observed that cells treated with metformin exhibited significantly lower mammospheres-forming efficiencies (MFE), also when exposed to TGFβ1. Moreover, the aforementioned effect of metformin on miRNAs expression, taken together with the results described in this paragraph, suggest that the drug inactivates crucial functions to CSCs survival [33,34,35,36].

## 4. Discussion

### 4.1. Summary of Evidence

The aim of this systematic review was to evaluate the regulation of expression of various miRNAs in CSCs, underlying the anti-cancer properties of metformin. Cancer treatment is a great challenge for medicine, therefore understanding the molecular basis of multidrug resistance, metastasis or tumor relapse is key to developing new therapies with better therapeutic outcomes for oncology [24,28]. One potential way to treat cancer is to use agents that directly affect CSCs functions. CSCs have the capacity of self-renewal and differentiation potential; thus, they can contribute to cancer therapy resistance, metastasis and tumor relapse [18]. There are many transcription factors (Oct 4, Sox 2, Nanog, KLF4, MYC) or signaling pathways (Wnt/β-catenin, Notch, Hh, NF-κB, JAK-STAT, TGF/Smad, PI3K/AKT/mTOR, PPAR) that are crucial in CSCs regulation. However, it is not fully understood how molecular mechanisms of CSCs are regulated [24].

In recent years, several miRNAs have been connected to anti-cancer mechanisms [37]. Moreover, down-regulation of some miRNAs was observed in tumors. Accordingly, miRNAs may affect major CSCs functions that lead to better outcomes of cancer patient treatment [20,28,37]. In this systematic review, researchers examined changes in expression of various types of miRNAs in CSCs listed below: miR-708, miR-27b, let-7a, let-7b, miR-101, miR-200b, miR-200c, miR-26a miR-181a and miR-96 [33,34,35,36]. miR-708 has been considered a cancer development suppressor in various types of cancers [38]. Previous studies showed that miR-708 overexpression led to decreased tumorigenesis through, for example, inhibition of cellular FLICE-like inhibitory protein (c-FLIP) [39], SMAD family member 3 (SMAD3) [40], zinc finger E-box-binding homeobox 1 (ZEB1) [41] or CD47 [42]. miR-27b is known for its dichotomous role in tumorigenesis. It has been reported that expression of miR-27b is increased in triple negative breast cancer [43,44]. On the other hand, miR-27b may act as suppressor gene in gastric cancer proliferation and metastasis by suppressing nuclear receptor subfamily 2 (NR2F2) [45]. Other miRNAs that have been found to be downregulated in cancers are the let-7 family. Mostly, let-7 are regulators of cell differentiation—downregulation of let-7 is a marker of less differentiated cancer [46,47]. Moreover, let-7 are linked to immunotherapy in various cancers through regulation of Toll-like receptors [48]. Various studies reported that miR-26a acts as a tumor suppressor by downregulating c-MYC pathway [49], cAMP regulated phosphoprotein 19 (ARPP19) [50], HOXC9 [51]. Other aforementioned miRNAs have also been linked to cancer suppression by regulating proliferation, apoptosis, metastasis and angiogenesis: miR-101 targets STMN1 [52], EZH2 [53]; miR-200 family members targets ZEB1 and SIP1 [54,55]. It must be noted that many miRNAs show a dichotomous role in tumorigenesis, besides the above mentioned miR-27b, for example, miR-181a [56] and miR-96 [57,58]. Published articles that have been analyzed in this systematic review focused on miRNAs expression in breast cancer [33,34,36] and pancreatic cancer [35]. Tan et al. [33] showed that expression of miR-708 was down-regulated in BCSCs. It has been reported that miR-708 regulates self-renewal capacity, phagocytosis and chemosensitivity in breast cancer. In addition, CD47 was identified as a direct target of miR-708 [33]. CD47 is a cell surface protein and its roles are crucial in immune system function and tumorigenesis. Prior studies have showed that CD47 is overexpressed in many hematopoietic and solid tumors and it correlates with worse clinical prognosis [59]. Takahasi et al. [34] identified gene encoding ENPP1 as a direct target of miR-27b that acts a tumor suppressor of breast cancer cells. Authors showed that miR-27b regulates the generation of an SP fraction that was linked to docetaxel resistance [34]. ENPP1 promotes the expression and cell surface localization of ABCG2 which is involved in the development of multidrug resistance, for example, in breast cancer, esophageal cancer, lung cancer [34,60,61,62]. Moreover, ENPP1 was reported as a promoter of generation of the SP fraction through upregulation of ABCG2 mRNA. ABCG2 regulates efflux activity of SP fraction that includes efflux of anticancer drugs [34]. In addition, Takashi et al. [34] reported that SP fraction was generated from miR-27b downregulated luminal-type breast cancer cells. Oliveras-Ferraros et al. [36] have identified let-7a downregulated expression in breast cancer cells that led to dedifferentiation and self-renewal capacity of cells. Moreover, TGFβ1 was found to upregulate miR-181a and downregulate miR-96 in breast cancer cells [36]. Previous studies confirmed that TGFβ1 induce sphere formation through upregulating miR-181a [63,64]. Bao et al. [35] showed that miR-26a plays a key role in the regulation of EZH2 and EpCAM mRNAs and proteins. Re-expression of miR-26a decreased the expression of EZH2 and EpCAM proteins and EZH2, Oct4¸ Notch-1 and EpCAM mRNAs [35]. EZH2 is the catalytic subunit of the polycomb repressive complex 2 (PRC2) and acts as lysine methyltransferase that is involved in the epigenetic regulation of gene transcription—methylation of histone H3 [65,66]. It has been shown that EZH2 is overexpressed in many tumors, such as breast cancer, ALL, Burkitt lymphomas, and is associated with poor clinical prognosis [35,65]. Previous data revealed that miR-26a and miR-101 could downregulate EZH2 which decreases self-renewal capacity and induces apoptosis in cancer cells [53,67].

**Figure 2:**
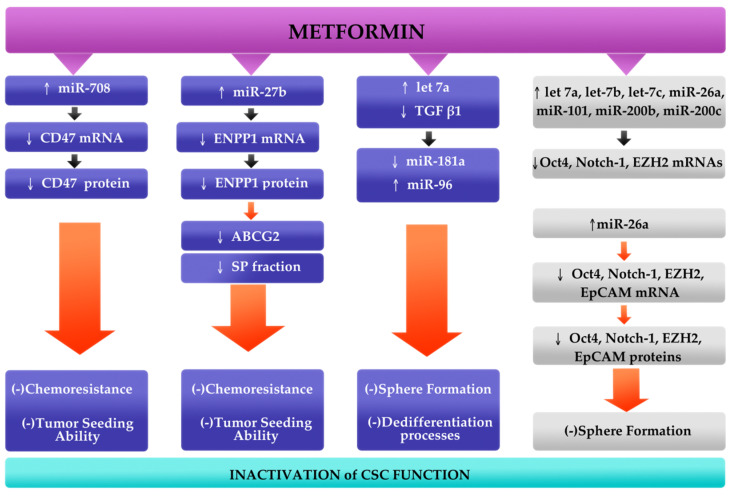
Conceptual mechanism of action of metformin. It has been reported that metformin upregulates miR-708, miR-27b and let-7a in breast cancer (**blue blocks**), and let-7 family, miR-200 family, miR-101 and miR-26a in pancreatic cancer (**gray blocks**). Metformin, through its ability to downregulate major cancer stem cells (CSCs) marker genes (CD47, ENPP1, EZH2, EpCAM, Oct4, Notch-1), acts as an anti-tumor agent that leads to suppression of chemoresistance, sphere formation and dedifferentiation processes and tumor seeding ability [33,34,35,36].

It has been presumed that metformin could block tumorigenesis by inactivation of CSCs. Various studies demonstrated that the mechanism of action of metformin is associated with AMPK/mTOR and insulin/IGF-1, MAPK and NF-κB signaling pathways [68,69]. Therefore, metformin antitumor effects are based on activation of AMPK or inhibition of mTOR [68]. It has been shown that metformin treated cancers exhibit antiproliferative effects, increased chemosensitivity, enhanced angiogenesis and prolonged tumor remission [69,70]. Thus, it appears that through the aforementioned pathways, metformin could inhibit self-renewal capacity, proliferation, migration and invasion of the CSCs [28,69]. However, the molecular mechanism of action of metformin remains unclear. One of the possible explanations is the modulation of various miRNAs expression that leads to major changes in functions of CSCs. As described above, studies included in this article demonstrated that CSCs could be eradicated by re-expression of miRNAs [33,34,35,36]. Moreover, all analyzed miRNAs were upregulated when metformin was added. Metformin modulates the following axes: miR-miR-708/CD47 in breast cancer [33], miR-27b/ENPP1 in breast cancer [34], 26a/EZH2 in pancreatic cancer [35], and blocks TGFβ1-induced upregulation of miR-181a and downregulation of miR-96 in breast cancer [36]. In addition, metformin also upregulates let-7 family, miR-200 family, miR-101 and Oct4, Notch-1, and EZH2 mRNAs in pancreatic cancer cells [35]. It has been showed that metformin inhibited sphere formation that suggests its major role in the inhibition of self-renewal capacity of CSCs [35,36]. All four studies analyzed reported positive effects of metformin in attenuating major CSCs functions through regulation of miRNAs expression (Figure 2) [33,34,35,36].

### 4.2. Limitations

This is the first systematic review that shows metformin effects on CSCs through regulation of expression of various miRNAs. There are limitations to this paper: included studies were performed on the cell lines but not on organisms, studies examined different miRNAs that make it impossible to analyze statistically, only two studies showed the effect of metformin on sphere formation and low number of studies were included. Therefore, performance of further investigations and clinical trials are required in order to bring a better understanding of the mechanism of action of metformin and the functions of CSCs.

## 5. Conclusions

In conclusion, this systematic review reports that metformin could inhibit tumorigenesis via targeted eradication of CSCs. The aforementioned studies show another possible mechanism of action of metformin which involves miRNAs. It is of great interest to fully and precisely understand the molecular role of metformin in the regulation of miRNAs. The above described preclinical studies implicate that metformin may improve therapeutic outcomes of breast and pancreatic cancer patients. However, functions of CSCs are still not fully understood and more studies are needed to examine CSCs molecular role in tumorigenesis. Apart from performing clinical trials on cancer patients, other areas of investigation may help in precisely understanding metformin-miRNA-CSC pathway. TCGA (The Cancer Genome Atlas) gave a better understanding of the genetic basis of the cancer through analyzing the genome. Therefore, computational tools may be useful in describing the molecular mechanism of action of metformin and its impact on miRNAs and CSCs. To sum up, further investigation is needed.

## Figures and Tables

**Table 1:** Reporting quality scheme.

		The Presence of the Information about Study Design		
Reporting quality	Is the cell origin and cell type used reported?	Reported	Not clearly reported	Not reported
	Is the dose of exposure reported?	Reported	Not clearly reported	Not reported
	Is the time of exposure reported?	Reported	Not clearly reported	Not reported

**Table 2:** Reporting the risk of bias scheme.

		The Presence of the Information of the Risk of Bias (Yes/No)		Risk Unknown
Performance bias	Was the exposure randomized?	Yes	No	Not reported
	Was the exposure blinded?	Yes	No	Not reported
	Have more than one cell lines been used?	Yes	No	-
Selection bias	Is the cell vitality scored/measured?	Yes	No	Not reported
	Were all measured outcomes reported?	Yes	No	Not reported
Detection bias	Were the experimental conditions the same for control and exposure treatment?	Yes	No	Not reported
Other bias	Was there no industry sponsoring involved?	Yes	No	Not reported

**Table 3:** Studies’ characteristics.

Author, Year	Study Design	Type of Cancer	Cell Lines	Animal	Intervention	miRNA	Main Outcomes
Tan et al., 2019 [33]	In vitro and in vivo	Breast cancer	MDA-MB-231, MCF-7	female BALB/c nude mice	Metformin	miR-708	Increased chemosensitivity and attenuated CSCs.
Takahashi et al., 2015 [34]	In vitro and in vivo	Breast cancer	MCF-7, ZR75-1, MDA-MB-231	female NON/SCID mice	Metformin	miR-27b	Increased chemosensitivity and inhibited tumor seeding ability in CSCs.
Bao et al., 2011 [35]	In vitro and in vivo	Pancreatic cancer	AsPC-1, AsPC-1-GTR, MiaPaCa-2, MiaPaCa-2-GTR	female CB17/SCID mice	Metformin	miR-26a; let-7; miR-200; miR-101;	Suppression self-renewal capacity, proliferation, migration and invasion in CSCs.
Oliveras-Ferraros et al., 2011 [36]	In vitro	Breast cancer	MCF-7	none	Metformin; Metformin + TGFβ1	let-7a; miR-181a; miR-96	Suppression TGFβ1 functions and dedifferentiation processes.

Cancer stem cells (CSCs); non-obese diabetic/severe combined immunodeficiency (NON/SCID); transforming growth factor β 1 (TGFβ1).

**Table 4:** Assessment of the quality of the included studies.

		Tan et al. [33]	Takahashi et al. [34]	Bao et al. [35]	Oliveras-Ferraros et al. [36]
Reporting quality	Is the cell origin and cell type used reported?	Reported	Reported	Reported	Reported
	Is the dose of exposure reported?	Reported	Reported	Reported	Reported
	Is the time of exposure reported?	Reported	Reported	Reported	Reported

**Table 5:** Assessment of the risk of bias of the included studies.

	**Was the Exposure Randomized?**	**Not Reported**	**Not Reported**	**Not Reported**	**Not Reported**
Performance bias	Was the exposure blinded?	Not reported	Not reported	Not reported	Not reported
	Has more than one cell line been used?	Yes	Yes	Yes	No
Selection bias	Is the cell vitality scored/measured?	Yes	Yes	Yes	Yes
	Were all measured outcomes reported?	Yes	Yes	Yes	Yes
Detection bias	Were the experimental conditions the same for control and exposure treatment?	Yes	Yes	Yes	Yes
Other bias	Was there no industry sponsoring involved?	Not reported	Yes	Yes	Not reported

**Table 6:** Analysis of miRNAs expression in tumor cells.

Author	Type of Tumor Cells	Type of miRNA	Target Expression	Effect of miRNA Regulation
Tan et al. [33]	breast cancer cells	miR-708	↓ CD47 mRNA and protein	Downregulation causes mammosphere formation. Upregulation induces sensitivity of cancer cells to drug therapy.
Takahasi et al. [34]	breast cancer cells	miR-27b	↓ ENPP1 mRNA and protein	Downregulation causes formation of SP fractions that leads to drug resistance. Upregulation inhibits the expression of ABCG2 transporter by suppressing ENPP1.
Bao et al. [35]	MiaPaCa-2	miR-26a	↓ EZH2, EpCAM proteins and mRNAs	Upregulation causes decrease in the formation of pancreatospheres.
	MiaPaCa-2 tumor sphere		↓ EZH2, Oct4, Notch-1, EpCAM mRNAs	

↓—downregulation; ATP-binding cassette super-family G member 2 (ABCG2) transporter; ectonucleotide pyrophosphatase/phosphodiesterase 1 (ENPP1); epithelial cell adhesion molecule (EpCAM); enhancer of zeste homolog 2 (EZH2); side-population cells (SP fraction).

**Table 7:** Influence of metformin on expression of miRNAs, mRNAs and other molecules.

Author	Type of Cells	Dose	Control	Time	Expression of miRNA	Expression of mRNA	Expression of Other Molecules
Tan et al. [33]	MCF-7.SC, MDA-MB-231.SC	10 (mM) Met	PBS	48 h	↑ miR-708	↓ CD47	-
	MCF-7.SC anti-miR-708, MDA-MB-231.SC anti-miR-708	0.3, 1.0, 3.0 (mM) Met	DMSO, β-actin (loading control)	72 h	-	-	↓ CD47 protein
Takahasi et al. [34]	MCF-7 co-transferred with pTK-GLuc027bs and pSV40-CLuc	0.1, 1.0, 10.0, 100.0 (mM)	0 (mM) Met	48 h	↑ miR-27b	-	-
	MCF-7-luc anti-miR-27b-DR, ZR75-1-luc anti-miR-27b	0.1, 0.3, 1.0, 3.0, 10.0 (mM)	DMSO, β-actin (loading control)	72 h	-	-	↓ ENPP1 protein
Bao et al. [35]	Pancreatospheres of pancreatic cancer cells	20 (mM) Met	0 (mM) Met	1 w	↑ let-7a, let-7b, let-7c, miR-26a, miR-101, miR-200b, miR-200c	↓ Oct4, Notch-1, EZH2, Nanog *	-
	Secondary pancreatospheres of mouse xenograft tumor derived from MiaPaCa-2 sphere-forming cells	20 (mM) Met	0 (mM) Met	1 w	-	-	↓ CD44, EpCAM proteins
Oliveras-Ferraros et al. [36]	MCF-7	1, 10 (mM); 1, 10 (mM) + 100 (ng/mL) TGFβ1	0 (mM) Met, 0 (ng/mL) TGFβ1	48 h	↑ let-7a, miR-96, ↓ miR-181a, miR-183	-	-

↑—upregulation; ↓—downregulation; dimethyl sulfoxide (DMSO); ectonucleotide pyrophosphatase/phosphodiesterase 1 (ENPP1); epithelial cell adhesion molecule (EpCAM); enhancer of zeste homolog 2 (EZH2); metformin (Met); phosphate-buffered saline (PBS); transforming growth factor β 1 (TGFβ1); * Nanog mRNA relative expression was only decreased in pancreatospheres of MiaPaCa-2 and MiaPaCa-2-GTR cells.
